# Acute Necrotizing Pancreatitis Leading to Hemosuccus Pancreaticus and Hemorrhagic Shock in the Setting of Decompensated Cirrhosis

**DOI:** 10.7759/cureus.75111

**Published:** 2024-12-04

**Authors:** Joseph Delly, Sevag Hamamah, Faizi Hai

**Affiliations:** 1 Department of Internal Medicine, Scripps Mercy Hospital, San Diego, USA; 2 Department of Gastroenterology, Scripps Mercy Hospital, San Diego, USA

**Keywords:** hemorrhagic shock, hemosuccus pancreaticus, necrotizing pancreatitis, pseudoaneurysm, upper gastrointestinal bleed

## Abstract

Hemosuccus pancreaticus (HP) is a rare, life-threatening cause of upper gastrointestinal bleeding, often linked to chronic pancreatitis and pseudoaneurysm rupture into the pancreatic duct. However, its occurrence in acute necrotizing pancreatitis with decompensated cirrhosis is exceedingly rare and poses significant diagnostic and treatment challenges. We report a case of a 34-year-old male with decompensated alcoholic cirrhosis who developed hemorrhagic shock from HP following acute necrotizing pancreatitis. The initial imaging revealed a pancreatic tail hematoma and a splenic artery pseudoaneurysm, that was later found to have ruptured into the pancreatic duct, causing intermittent GI bleeding. Endoscopy showed clots extruding from the ampulla, and angiography confirmed active bleeding, leading to endovascular coil embolization. Despite intervention, the patient's coagulopathy and hemodynamic instability, related to his cirrhosis, worsened, ultimately resulting in death under comfort care. This case underscores the importance of considering HP in patients with pancreatic disease and unexplained GI bleeding, especially in the presence of pseudoaneurysms, as timely endovascular or surgical management, coupled with a multidisciplinary approach, is essential to improve outcomes.

## Introduction

Hemosuccus pancreaticus (HP) is a rare yet recognized cause of upper gastrointestinal (GI) bleeding, with delayed diagnosis often contributing to significant mortality [1]. Chronic pancreatitis is the most common risk factor (60-65% of cases), while less common causes include peripancreatic arterial pseudoaneurysms (10-20%), pancreatic pseudocysts (10-20%), and acute pancreatitis (5-10%) [1-3]. The pathophysiology of HP involves chronic enzymatic autodigestion of peripancreatic vessels, which leads to aneurysmal formation and rupture, resulting in bleeding through the pancreatic duct [4]. Rupture often involves major peripancreatic arteries, including the splenic, pancreaticoduodenal, and gastroduodenal arteries [5]. Notably, HP accounts for only 1 in 1,500 cases of upper gastrointestinal bleeds [2]. In most cases, bleeding is usually intermittent and presents as abdominal pain, GI bleeding, and anemia, but severe cases can cause hemorrhagic shock and rapid bleeding into the duodenum, with mortality rates around 10%, higher in males (7:1 ratio) [6].

Diagnosis is challenging due to coexisting conditions common in GI bleeding, such as cirrhosis, critical illness, and alcohol use [7-9]. Despite the low index of suspicion for HP due to its rarity, HP should be considered in individuals with pancreatic disease and unexplained or recurrent GI bleeding [1]. Acute GI hemorrhage should prompt suspicion for HP in cases with peripancreatic arterial pseudoaneurysms, pancreatic hematomas, or acute necrotizing pancreatitis, with alcohol use adding further complexity given its overlap with other etiologies of upper GI bleeding [7,10].

We present a case of HP and hemorrhagic shock in a 34-year-old with acute necrotizing pancreatitis and decompensated alcoholic cirrhosis. Initially, the patient presented with altered mental status and melena. Computed tomography (CT) angiography revealed a pancreatic tail hematoma and pancreatic head calcification, without evidence of aneurysm or necrosis. After several days, further imaging revealed a peripancreatic pseudoaneurysm and enlarging pancreatic tail hematoma amid acute onset necrotizing pancreatitis. Esophagogastroduodenoscopy (EGD) showed large clots and bleeding through the ampullary duct, leading to hemorrhagic shock and a worsening prognosis. 

This case highlights HP as a severe complication of acute necrotizing pancreatitis, demonstrating the diagnostic challenges in patients with cirrhosis and GI bleeding, as well as the importance of a multidisciplinary approach in managing HP-associated hemorrhagic shock. The case also addresses the prognostic implications in patients with pancreatitis-related vascular complications, particularly in those with end-stage liver disease.

## Case presentation

**Figure 1:**
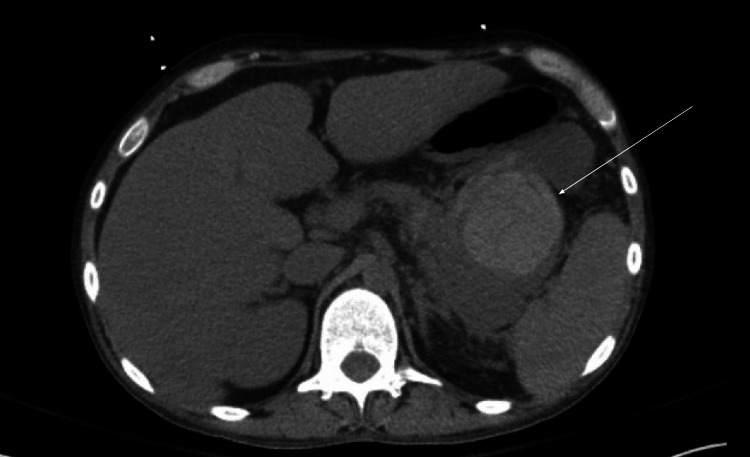
CT abdomen and pelvis on hospital day 1 CT demonstrating a 7.5 cm hyperdense mass-like lesion in the lesser sac, consistent with hemorrhage abutting the stomach, pancreatic tail, and spleen. No evidence of acute pancreatitis or necrotizing process was observed.

A 34-year-old male with a history of alcoholic cirrhosis (MELD 3.0 score of 30) presented after being found unconscious following heavy alcohol use, along with intermittent episodes of melena. The initial laboratory evaluation revealed elevated aspartate aminotransferase (AST), alanine transaminase (ALT), and total bilirubin with associated leukocytosis, normocytic anemia, elevated lipase, lactic acidosis, and acute kidney injury. On exam, he had encephalopathy, jaundice, scleral icterus, and mild, non-tender abdominal distention. A CT angiogram of the abdomen revealed a 6.2 cm hematoma anterior to the pancreatic tail, extending into the splenic hilum and abutting the greater curvature of the stomach, along with possible pancreatitis and splenic vein thrombosis (Figure 1).

He required multiple packed red blood cell (pRBC) transfusions for suspected ongoing GI bleeding and treatment for alcohol withdrawal. Initial EGD demonstrated LA grade D esophagitis, no esophageal varices, mild portal hypertensive gastropathy, and mild extrinsic compression of the greater gastric curvature from the hematoma. Magnetic resonance cholangiopancreatography (MRCP) revealed a 6.9 cm pancreatic tail mass with possible pancreatic duct dilation. A follow-up CT confirmed a 6.5 cm pancreatic tail hematoma, an 18 mm splenic artery pseudoaneurysm, nonocclusive splenic vein thrombosis, and suspicion of necrotizing pancreatitis. Antibiotics were initially withheld due to the absence of signs of active infection.

**Figure 2:**
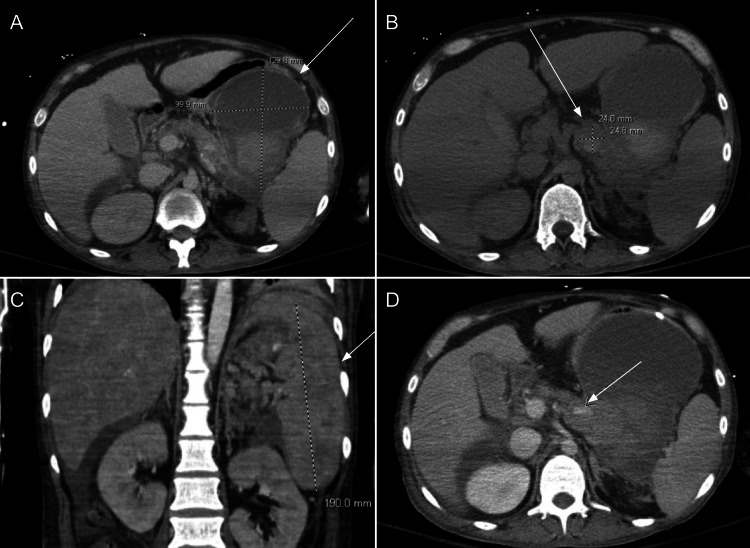
CT abdomen and pelvis with and without contrast on day 13 of hospitalization demonstrated a pancreatic tail hematoma and pseudoaneursym CT abdomen and pelvis with and without contrast on day 13 of hospitalization showed an enlarging pancreatic tail hematoma (10 cm width, 19 cm height, 13 cm length) (A and C). A 2.5 cm pseudoaneurysm in the pancreatic body had slightly increased in size with a developing thrombus that opacifies approximately 40% of the pseudoaneurysm (B). Hypodensity in the pancreatic tail region is consistent with acute necrotizing pancreatitis (D).

A repeat CT on day 13 of hospitalization confirmed necrotizing pancreatitis, an enlarged 2.5 cm splenic artery pseudoaneurysm, and a significantly increased pancreatic tail hematoma (10 cm x 19 cm x 13 cm) (Figure 2). The patient developed worsening ascites, confirmed as portal hypertensive ascites without spontaneous bacterial peritonitis (SBP), followed by an episode of hematochezia. Urgent CT angiography showed no active extravasation but noted a stable lesser sac hematoma and persistence of splenic artery pseudoaneurysm.

On day 18 of hospitalization, during preparation for a colonoscopy, the patient developed fever, tachycardia, and hypotension, requiring intensive care unit (ICU) transfer and vasopressor support. He was started on vancomycin and piperacillin-tazobactam for suspected septic shock secondary to necrotizing pancreatitis, alongside hemorrhagic shock from GI bleeding.

**Figure 3:**
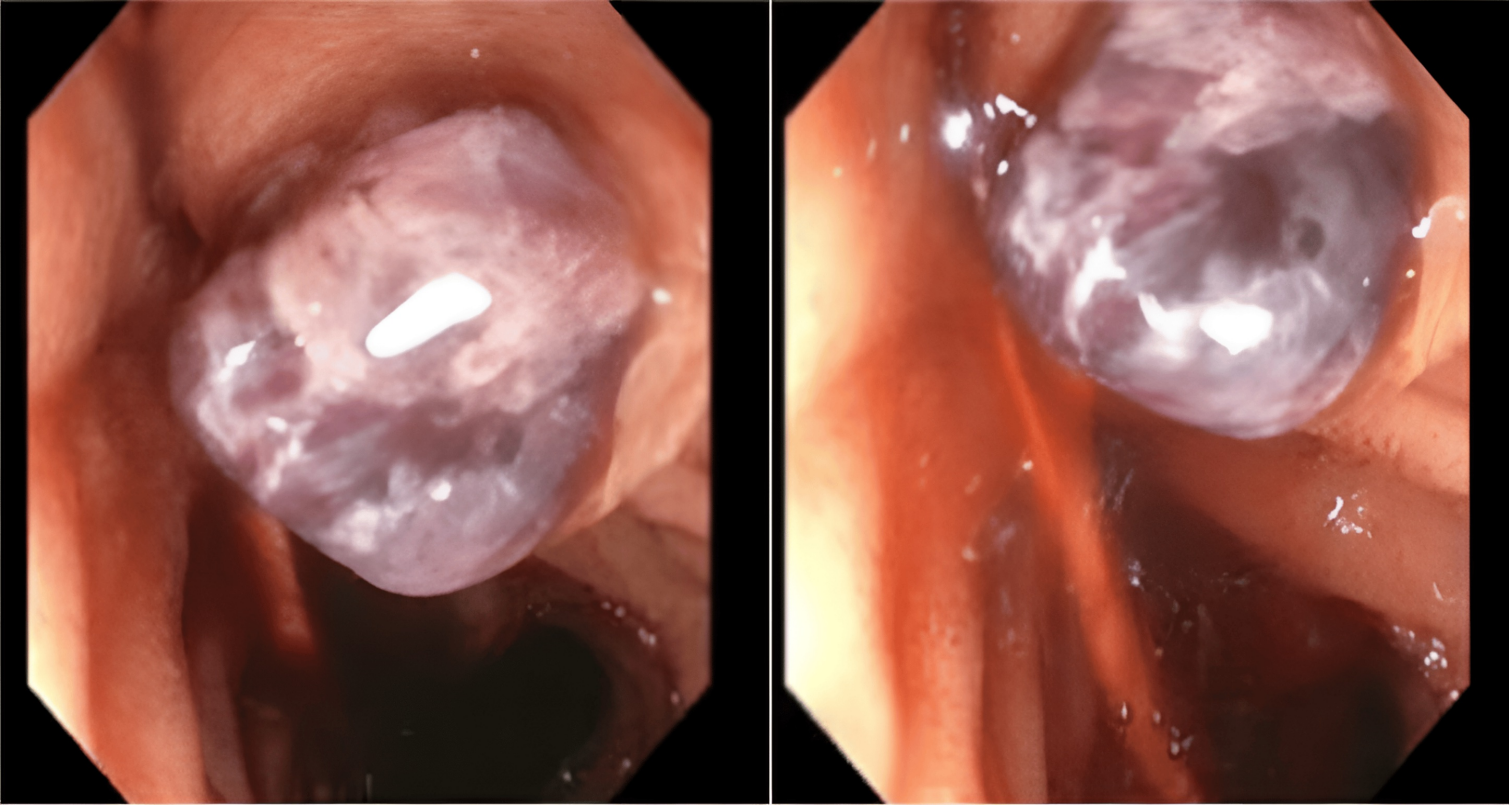
Endoscopic images of fresh blood and clots protruding from the ampulla of Vater into the second part of the duodenum Endoscopic images showing fresh blood and clots protruding from the ampulla of Vater into the second part of the duodenum. Images were taken using a side-viewing duodenoscope.

Repeat EGD revealed a small distal esophageal varix, moderate portal hypertensive gastropathy, and blood with clots in the duodenum. A side-viewing duodenoscope revealed a large clot extruding from the ampulla of Vater, consistent with HP (Figure 3). Compression of the greater curvature of the stomach by the expanding pancreatic tail hematoma was also noted.

**Figure 4:**
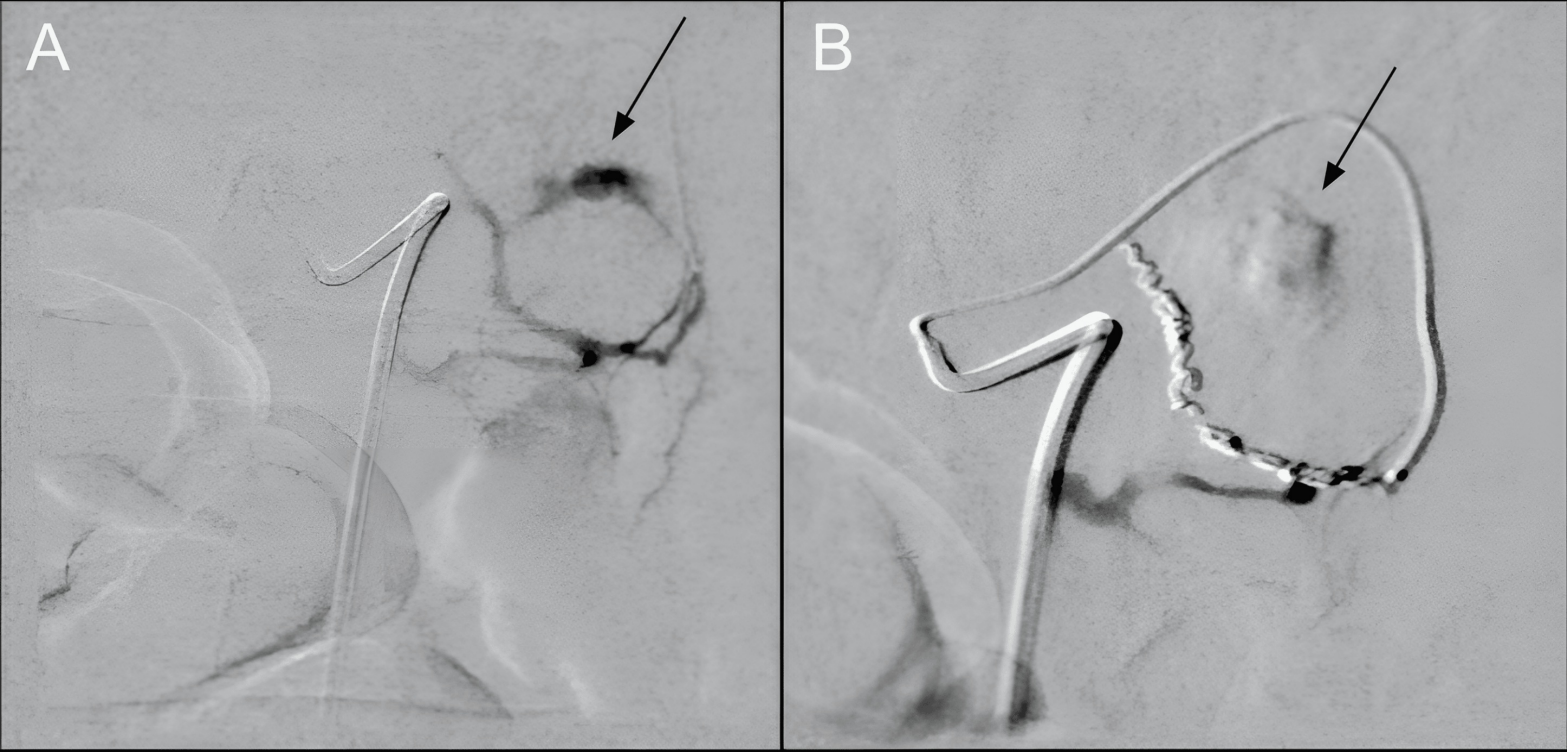
Pseudoaneurysm with active arterial extravasation arising from a small pancreatic arterial branch, successfully treated with coil embolization Angiogram of the pancreatic arterial branch and its secondary/tertiary branches demonstrated a pseudoaneurysm with active arterial extravasation originating from a small pancreatic arterial branch (A). The branch was successfully treated with coil embolization, with no subsequent evidence of extravasation (B).

Interventional radiology (IR) was consulted and diagnostic angiography was performed on the peripancreatic vasculature, identifying a pseudoaneurysm involving an unnamed pancreatic perforator artery from the splenic artery, anastomosed with the pancreatica magna artery. Initial attempts to embolize were unsuccessful due to prominent arterial spasms; however, a repeat attempt showed active arterial extravasation seen from a small pancreatic arterial branch that was successfully treated with coil embolization (Figure 4). 

The antibiotic regimen was escalated to meropenem for three days for suspected ongoing infection. Despite the resolution of septic and hemorrhagic shock, persistent hematochezia necessitated near-daily pRBC transfusions. A repeat CT angiography revealed a 7 mm residual pseudoaneurysm. IR advised against repeat angiography or embolization due to the development of multiple aneurysms. General surgery was consulted, and he was deemed a poor surgical candidate due to his decompensated cirrhosis. Given the patient's deterioration and risks of complications, a goals-of-care discussion held with the patient and family led to the decision to not escalate medical care.

The patient’s hematochezia and hepatic encephalopathy worsened. Therapeutic paracentesis was performed for comfort due to worsening ascites. A few days later, he developed severe shock, likely secondary to acute GI bleeding, along with profound hypoxia with altered mental status. The patient's family at this time elected for comfort care measures, and he passed away shortly thereafter. 

## Discussion

This case presents a unique presentation of HP secondary to acute necrotizing pancreatitis, resulting in hemorrhagic shock in a patient with decompensated cirrhosis. HP with coexisting cirrhosis is exceptionally rare, with only a few reports documenting this combination [7,11-13]. In a retrospective study at a single center, only 1 out of 17 patients treated for HP had cirrhosis [14]. Most documented cases attribute intermittent GI bleeding in HP to a pseudoaneurysm from chronic pancreatitis, with rare instances of severe bleeding or hemorrhagic shock [7,11-13].

Notably, no previous reports associate HP with acute necrotizing pancreatitis, making this case significant as the first to link HP, severe hemorrhagic shock, and death with end-stage liver disease. Our patient’s severity was compounded by a pancreatic pseudocyst, arterial pseudoaneurysm, and a pancreatic tail hematoma, emphasizing the complexity of managing HP in patients with cirrhosis, where treatment options are often limited. This case contributes to the growing literature on HP, emphasizing the need to promptly recognize and manage comorbidities to enable timely diagnosis and intervention, potentially preventing fatal outcomes. 

Pathophysiological links between hemosuccus pancreaticus, cirrhosis, and acute necrotizing pancreatitis

In this case, the patient’s decompensated cirrhosis (MELD 30, Child Pugh C) significantly worsened the prognosis, as cirrhosis-mediated portal hypertension and coagulopathy amplified the risk of severe hemorrhage [13]. In cirrhosis, elevated pressures in the portal venous system, including the splenic and mesenteric veins, drive collateral formation, indirectly promoting the formation of pseudoaneurysms [15,16]. Splenic vein congestion or thrombosis increases stress on local vasculature, including the splenic artery [17-19]. Splenomegaly, resulting from venous congestion, places extrinsic pressure on the splenic artery, raising the likelihood of aneurysms, as shown in studies linking enlargement of the splenic vein with splenic artery aneurysm [20-22]. Chronic elevation in portal pressure, leading to the formation of collateral circulation, further increases hemodynamic demand on the arterial system. This compensatory hyperdynamic circulation places increased strain on arterial walls, resulting in localized vessel weakening and dilation [16,22]. 

Cirrhosis-associated coagulopathy also worsened the patient’s condition, as impaired clotting factor synthesis, vitamin K deficiency, and platelet sequestration from splenomegaly elevated the risk of bleeding [23]. Overactive fibrinolytic pathways, in addition to vessel fragility, increase susceptibility to severe hemorrhage in acute necrotizing pancreatitis [24].

Vascular involvement occurs early in acute pancreatitis in up to 25% of cases, increasing to 43% of cases in necrotizing pancreatitis, manifesting as venous phlebitis, arteritis, venous thrombosis, or pseudoaneurysms [25]. Hemorrhage occurs in 3-6% of necrotizing pancreatitis cases, with over half due to pseudoaneurysmal rupture [26-28]. In this case, we propose that acute necrotizing pancreatitis triggered a rupture in a pancreatic arterial branch of the splenic artery, leading to hemorrhagic via HP. Bleeding typically occurs in the retroperitoneum, but in cases like this, blood may flow through the pancreatic duct into the gastrointestinal tract [25, 29].

Necrotizing pancreatitis results in HP through mechanisms such as vascular erosion, tissue necrosis, and pseudocyst formation [30]. Pancreatic enzymes induce autodigestion, eroding vessels and promoting pseudoaneurysms, which develop in approximately 4% of necrotizing pancreatitis cases, with a mortality of up to 14% [31]. The splenic artery is most affected (38%), followed by the gastroduodenal and pancreaticoduodenal arteries [4,5]. Pancreatic tissue necrosis compromises the integrity of surrounding vasculature, facilitating hemorrhage in the acute setting [32, 33]. Pancreatic pseudocysts may also exacerbate HP by compressing and rupturing nearby vessels, where compromised vasculature leads to rapid GI hemorrhage [34].

Diagnosis and treatment of hemosuccus pancreaticus

HP is a challenging diagnosis due to its anatomic complexity and poor visualization of the pancreaticobiliary system during endoscopy [35]. Intermittent bleeding further complicates detection, as endoscopy may fail to detect HP if active bleeding is absent [35,36]. Studies report that upper endoscopy identifies HP in only ~30% of cases, with improved success using a side-viewing duodenoscope for ampulla visualization [1]. Negative endoscopy does not rule out HP, as other causes of bleeding like hemobilia and ampullary tumors remain in the differential diagnosis [1,37].

In our case, the initial EGD demonstrated esophagitis and portal hypertensive gastropathy, without ampullary bleeding. Subsequent imaging revealed a pseudoaneurysm and an enlarging pancreatic tail hematoma, leading to repeat endoscopy and observation of clots protruding from the ampulla using a side-viewing duodenoscope. Diagnosis often requires multi-modal imaging, with contrast-enhanced CT used to detect pseudoaneurysms, pseudocysts, and extravasation [38]. Angiography of peri-pancreatic vessels enables both diagnosis and treatment through embolization [38].

Angiographic embolization is currently the first-line treatment for HP from pseudoaneurysmal bleeds and has seen technical success rates of 97%, with long-term success rates around 82-88% [5,14,38]. Technological advances have improved outcomes considerably over the past decade [39]. In cases of concurrent cirrhosis, the management of HP is multidisciplinary, requiring collaboration amongst gastroenterology, interventional radiology, surgery, and critical care physicians. For initial stabilization, crystalloid fluids, blood transfusions, and vasopressors are crucial for achieving hemodynamic stability until definitive intervention is performed [40].

In cirrhotic patients, management includes coagulopathy correction using vitamin K, fresh frozen plasma (FFP), and platelets [41,42]. Vasoactive agents such as octreotide or terlipressin reduce portal pressure, alleviating strain on collateral vessels, while antibiotics prevent bacterial translocation and spontaneous bacterial peritonitis [43,44]. 

When coil embolization is unsuccessful, surgical hemostasis may be necessary to prevent exsanguination, with procedural options including distal or central pancreatectomy, splenectomy, or aneurysm ligation, depending on the bleeding location [6,45]. However, in cirrhotic patients with high Child-Pugh scores, surgical intervention has high operative mortality, especially in patients with Class C cirrhosis (up to 80%) [46]. For this patient, distal pancreatectomy might have been an option if embolization had failed but was contraindicated due to advanced cirrhosis [46,47]. Effective long-term management, which includes nutritional support, pancreatic enzyme replacement, and management of pancreatic pseudocysts or vascular abnormalities, remains critical for improving patient outcomes and long-term survival.

Recent advancements in treatment and the role of endoscopic ultrasound

Recent advancements in endoscopic technology have highlighted the utility of contrast-enhanced endoscopic ultrasound (EUS) for diagnosing and managing HP. EUS, particularly with contrast enhancement, provides a detailed view of vascular structures surrounding the pancreas, allowing for precise localization of pseudoaneurysms responsible for bleeding [48-50]. While non-contrast EUS is valuable in delineating pancreatic anatomy, it lacks the sensitivity needed to identify small, bleeding culprit vessels within the pancreatic tissue [50]. Contrast enhancement overcomes this limitation, making EUS a suitable option even for patients with renal impairment, as it avoids nephrotoxic contrast agents used in CT angiography. 

A recent case report describes successful EUS-guided treatment in two cases of HP using cyanoacrylate injection into the pseudoaneurysm, resulting in hemostasis and no recurrence over follow-up periods of three and six months [51]. This outcome highlights the potential role of EUS not only for diagnosis but also as a minimally invasive therapeutic intervention. Although underreported, EUS-guided management of HP may emerge as a critical intervention for managing pseudoaneurysmal bleeding in HP, particularly when traditional imaging and treatments pose greater risks or fail to control bleeding effectively.

## Conclusions

To summarize, acute necrotizing pancreatitis can lead to severe vascular complications, including pseudoaneurysmal rupture, ultimately resulting in HP. The mortality associated with pseudoaneurysmal rupture is high in cases of massive hemorrhage and hypovolemic shock. In patients with decompensated cirrhosis, underlying coagulopathy and hemodynamic changes exacerbate bleeding and further worsen outcomes. 

Therefore, early identification and intervention through endovascular coiling or surgical intervention is paramount for survival, alongside fluid resuscitation, and blood transfusion. While coil embolization remains the gold standard for endovascular intervention, newer therapies such as contrast-enhanced endoscopic ultrasound (EUS) are emerging as promising diagnostic and therapeutic tools. Ultimately, a multidisciplinary approach is essential for improving outcomes, particularly in navigating complex cases.
